# Failure to Treat Torsades de Pointes

**DOI:** 10.4021/cr139w

**Published:** 2012-01-20

**Authors:** Payam Yazdan-Ashoori, Genevieve Digby, Adrian Baranchuk

**Affiliations:** a Cardiology Division, Kingston General Hospital, Kingston, Ontario, Canada

**Keywords:** Torsades de Pointes, Temporary pacemaker, QT prolongation

## Abstract

A healthy 22 year old male with no history of cardiac disease was admitted with severe community acquired pneumonia that was initially treated with moxifloxacin and azithromycin. At admission, he was found to be hypokalemic and hypomagnesemic. Two days after admission, he experienced several episodes of Torsades de Pointes (TdP). He was initially treated with isoproterenol. A temporary transvenous pacemaker was inserted and set at a rate of 100 bpm. After correction of electrolytes, withdrawal of QT-prolonging medications and ventricular pacing at the mentioned heart rate, another episode of TdP ensued.We report and discuss a case of recurrent TdP in spite of conventional acute management for this condition.

## Introduction

Torsades de Pointes (TdP) is a polymorphic ventricular tachycardia that is often life-threatening if not properly detected and treated. Delayed repolarization by acquired or congenital mechanisms leading to long QT syndrome (LQTS) facilitates TdP. Therefore, treatment aims to correct and prevent the prolongation of the QT-interval, which can be brought about by electrolyte disorders, certain drugs, and/or bradycardia [1, 2].

We present a case of TdP that recurred despite acute management and discuss potential reason for failure of treatment.

## Case Report

**Figure 1:**
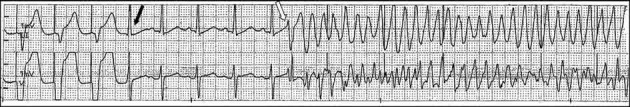
Temporary transvenous pacemaker set at 100 bpm. The patient’s intrinsic rate overrides the paced rate (black arrow). Four consecutives intrinsic beats at 115 bpm with a QTc-interval of 602 ms triggered recurrent TdP (white arrow).

A healthy, active 22 year old male with no history of cardiac disease presented to his family physician with several days of nausea, vomiting, cough, dyspnea and right-sided chest pain. He was prescribed moxifloxacin and naproxen, but after taking only one dose of each, he presented to the emergency department. He was admitted and treated for pneumonia with azithromycin 500 mg IV q24 h and moxifloxacin 400 mg IV q24 h. His initial potassium was 3.3 mmol/L. Two days later, the patient experienced what was thought to be a seizure-like episode. Later that same day, he suffered several runs of non-sustained TdP. At that point, isoproterenol was administered to increase the heart rate with the intention to shorten the QT-interval. His baseline heart rate was between 90 - 100 bpm. A right ventricular wire was inserted for temporary pacing at a rate of 100 bpm. Potassium (4.3 mmol/L) and magnesium (0.97 mmol/L) were replaced and antibiotics were switched to a non-QT prolonging agent. Despite this, another episode of TdP occurred seven hours after insertion of the temporary wire (Fig. 1). The temporary pacemaker was removed two days later, prior to transfer to our centre, as he was deemed stable.

Upon transfer, a right lower lobe empyema was drained, and the patient’s pneumonia was eventually treated with doxycycline and ceftriaxone. An exercise stress test did not reveal ST-segment changes and demonstrated normal QT behavior. The patient was discharged home. Tests for genetic disorders are pending at the time of preparation of this report.

## Discussion

This case highlights an example of TdP recurrence in the setting of acquired LQTS despite appropriate treatment. Our patient was managed by correcting his hypokalemia and hypomagnesemia, and by discontinuing potentially offending antibiotics. He was treated with temporary pacing with a transvenous ventricular wire.

Recommendations suggest initial treatment of TdP with a 2 g IV bolus of magnesium sulfate over 2 - 3 minutes followed by a 2 - 4 mg/minute infusion, independent of the initial magnesium concentration [3, 4]. The recommended target for potassium correction is usually around 4.5 - 5.0 mmol/L to prevent QT prolongation. Hypocalcemia (< 2.1 mmol/L) and hypomagnesemia (< 0.75 mmol/L) should be prevented as they also contribute to a lengthened QT-interval.

Offending medications should be immediately stopped. Isoproterenol and temporary pacing may be used emergently as a means to overdrive suppress the arrhythmia and prevent recurrence in addition to shortening the QT-interval and re-homogenizing ventricular refractory periods [4].

Unsuccessful treatment of TdP may result from several factors (Table 1). Firstly, the proper recognition of TdP is of paramount importance, as its management is different from similar arrhythmias such as ischemic polymorphic ventricular tachycardia (PVT) [5]. Misdiagnosis of TdP as PVT may lead to the use of amiodarone, which would exacerbate TdP, as previously demonstrated by our group [1]. Obviously, failure to discontinue QT prolonging drugs may result in TdP recurrence and perpetuate the arrhythmia. Similarly, correction of electrolytes including potassium, magnesium, and calcium would decrease the risk of recalcitrant TdP [6].

**Table 1:** Possible Causes for Failure to Treat TdP

Factor	Mechanisms
Maintenance of aggravating drugs	QT-interval prolongation
Uncorrected electrolyte disturbance	QT-interval prolongation
Ischemia	Increased dispersion of repolarization
Uncorrected bradycardia	QT-interval prolongation
Inadvertent failure of pacing by inappropriate rate setting	Intrinsic rate will prevail with no resolution of QT prolongation

Inadvertent ischemia is another possible reason for failure to treat TdP. Ischemia may lead to prolonged repolarization. It induces excessive dispersion of repolarization via differential shortening of the action potential in different layers of the myocardium, contributing to QT prolongation [7]. Increasing the rate in an attempt to overdrive suppress TdP will increase metabolic demand and aggravate the ischemic condition. The probability of ischemia in our case was low.

Bradycardia is also regarded as a risk factor for TdP. The initiation of TdP can involve a short-long-short sequence whereby an early after afterdepolarization (EAD) occurs after a relatively long R-R interval. It has been shown that chronic complete AV block leads to the down-regulation of repolarizing currents, thus prolonging repolarization reserve and the QT-interval [8]. A recent study by Subbiah et al. suggests that patients who develop TdP in the setting of AV block may in fact represent an 'unmasking' of latent congenital LQTS [9]. Genetic screening of patients with TdP and high-grade AV block may be considered. Bradycardia was not a major issue in the presented case; however, genetic testing was ordered to confirm the absence of a genetic predisposition to LQTS.

After considering the various factors that can lead to failure to treat TdP in this case, it became clear that the paced rate (100 bpm) was overridden by the patient’s intrinsic conduction. His intrinsic rate (115 bpm) overdrive suppressed the paced rate, and after 4 intrinsic beats with a prolonged QTc (602 ms), TdP recurred.

There is no consensus on the rate at which patients should be paced. Furthermore, although there is published experience on how to set up a permanent pacemaker (PPM) in TdP, there is no specific literature on temporary pacemakers. Pinski et al. reviewed 18 published cases of acquired LQTS and TdP in patients with PPMs. It was determined that none of the patients experienced TdP with an effective pacing rate >70 bpm [10].

In order to prevent TdP recurrence, a multifactorial approach that considers all aspects of this complex arrhythmia should be put in place. Failure to treat TdP usually involves at least one of the above mentioned factors. Their recognition and rapid correction may prevent further complications.

### Conclusion

This case represents recurrent TdP in the setting of appropriate textbook management. It highlights one of the circumstances that may be responsible for a failure to treat this condition; that is, inappropriate temporary pacing allowing the intrinsic rate to be faster than the pacing rate. An effective pacing rate to prevent TdP should be determined on a case-by-case basis in order to properly suppress intrinsic conduction.
